# AsanteTwiSenti: A Sentiment dataset of Ghanaian Asante Twi Tweets in a multilingual context

**DOI:** 10.1016/j.dib.2025.111460

**Published:** 2025-03-13

**Authors:** Mavis Sarah Gyimah, James Benjamin Hayfron -Acquah, Rose-Mary Mensah Gyening, Michael Asante, Umar Farouk Ibn Abdulrahman, Evans Kotei

**Affiliations:** Computer Science Department, Kumasi Technical University (KSTU), P.O. Box 854, Kumasi, Ghana

**Keywords:** Asante, Twi, Low-resource languages, Sentiment analysis, Multilingual, Ghanaian Pidgin, Ghanaian languages, Natural Language Processing

## Abstract

Ghanaian Asante Twi is the most widely spoken indigenous language in Ghana. It is a language of scholarship that is very rich in African studies and is taught in many universities across the globe. Despite its popularity, it lacks data resources in Sentiment Analysis, Named Entity Recognition, Part of Speech (POS) tagging, and in particular linguistic corpora. The paper introduces AsanteTwiSenti, a comprehensive sentiment corpus for the Ghanaian Asante Twi language with the methods and challenges encountered in the corpus construction. The AsanteTwiSenti corpus contains 10,095 tweets extracted from 30,507 tweets scraped from the Twitter API. Based on standard guidelines and data preprocessing, 8438 tweets are labeled as Positive, Negative, Neutral, Ghanaian-Pidgin, multilingual, and Monolingual. The AsanteTwiSenti corpus seeks to bridge the low-resource gap of the Twi Language, inspire the development of local Ghanaian language resources, and impact academic research of Asante Twi for Natural Language Processing(NLP), language preservation, and education.

Specifications Table

**Table:** 

Subject	Computing and Linguistics
Specific subject area	Natural Language Processing
Type of data	The AsanteTwiSenti corpus contains 8,438 annotated tweets from the 10,095 tweets collected from the Twitter API. It has the following labels: Positive, Negative, Neutral, Ghanaian-Pidgin, Multilingual, and Monolingual. The Ghanaian Pidgin and multilingual labels are added to create additional datasets for further research.
Data collection	Asante Twi Tweets containing sentiments are collected through the Twitter API. Twitter has extensive user-generated content which may not be related to a particular domain or topic [1]. These contents contain valuable information that may be subjective or objective and can be useful for research. Twitter may grant access to its Twitter Academic API upon application, Twitter has current and archived data and supports about 40 high-resource languages. Collecting data in these languages is easy however Twi Language is not on the list of supported languages; therefore, collecting tweets requires some effort. Similar to [2] approach, data is collected by querying the Twitter API using the following: **i. Twi stop words, emoji, and sentiment words:** The Twitter API is queried by using Twi words with stop words and emojis as key parameters. In Africa, different languages can have similar words, for example, the word “din” is found in Asante Twi and Hausa with different meanings, This introduces redundant data and noise on a language's dataset. To minimize this problem, tweets are collected using the location, longitude, latitude, and radius covering the area where the Asante Twi language is predominantly spoken in the West African sub-region. **i. Hashtags and Handles of people who tweet in the Asante Twi Language:** To be able to collect quality large-scale datasets, hashtags, and handles of people (#username and @user) is helpful, as opined by [3]. The queries are constructed to include hashtags and handles of people, entities, places, radio stations, TV programs, branded products, and services, The data collection stage aims to collect as many tweets as possible, i**i. Distant Supervision:** Emojis carrying sentiments, Positive, and Negative is used to query the Twitter API. Emojis add emotional signals that may be missing from conversations. Distant supervision with emojis is partially used to annotate the tweets. The use of emoticons and emojis as a distantly supervised method to classify tweets automatically into positive and negative classes has been used by [4,5]. By creating a dictionary of emojis with corresponding sentiment labels of positive and negative, if a tweet contains an emoji, a label is given based on the sentiment the emoji carries.
Data source location	The dataset is scraped from the Twitter API with the help of the Twarc Library and stored on a hard drive,.
Data accessibility	The datasets can be accessed from the GitHub Repository, Repository name: NLP Data identification number: 10.5281/zenodo.14506733 Direct URL to data: https://doi.org/10.5281/zenodo.14506733 Repository URL: https://github.com/msgyi/NLP as in [17]
Related research article	None

## 1 Value of the Data

• The advantages of a comprehensive corpus include closing the low-resource gap and aiding linguistic research, providing researchers with an avenue to study and analyze linguistic patterns. Asante Twi has folklore, proverbs, traditional Ananse stories, poems, palm wine tappers' songs, drumming, dancing, Kente and Adinkra designs that carry wisdom and wildlife tales that continue to inspire the moral character of Ghanaians.
• The study of a language can be compared to studying a species of a kind. Asante Twi is unique and is a medium to understand the world of the Asantes, and how words, phrases, and sentences in Twi are formed with ideas. The proposed corpus can empower speakers and improve understanding of the Twi language in areas like communication, translation services, dissemination of public information, and treating speech disorders.
• In education, the corpus is a useful resource that can support learners, teachers, and curriculum developers.
• Language death is a challenge currently, a corpus can help foster language safety and durability. This can impact other Ghanaian language research such that special attention is paid to language preservation, because the survival of a language is directly linked to the survival of a culture and history.
• The linguistic corpus will be a valuable resource for developing NLP tools and applications related to Asante Twi. Suitable language models can be built for machine language translation, question and answering, dictionaries, sentiment analysis, speech recognition, and other NLP tasks.

## 2 Background

The Akans in Ghana comprise Agona, Akuapem, Akwamu, Akyem, Asante, Bono, Fante, Kwahu, Wassa, and Sefwi tribal groups according to the Bureau of Ghana Languages (1996) in [16]. The Akan tribe of Ghana has mutually intelligible dialects of the Twi language. In Ghana, Ivory Coast, and some neighboring West African countries, different Twi dialects are spoken. The Akans are about 60 % of Ghana's population. Twi is an important Akan tribal language spoken by these tribes and [6] reports the Akan language as spoken predominantly among the indigenous languagescategory in Ghana.

Asante Twi is the dialect of the people of the Asante Region and it is the most widely spoken language in Ghana. It is linguistically rich, morphologically simple, and has a tonal system that affects word and phrase meanings. The language is prevalent, easy to learn, understand, and use in daily commerce across Ghana. In [7], the writers attest to the under-representation of African languages in Natural Language Processing(NLP). The challenge of scarce digital data resources such as the lack of appropriate keyboards, dictionaries and digital tools specific to African languages is a challenge for many under-resourced languages in Ghana. These languages receive less funding, expertise, and quality data because governments are not intentional about preserving their own languages. Social media content, however, provides an avenue to collect real data for research.

## 3 Data Description

The AsanteTwiSenti corpus contains 8438 annotated tweets. and the tweets are labeled using a multi-class system made available on the Light tag annotation tool [8] as Positive, Negative, Neutral, Ghanaian-Pidgin, multilingual, and Monolingual by annotators using standard guidelines. Positive, negative, and neutral classes are given to a tweet based on the sentiment carried by the tweet in the annotation process (Tables 1, 2).

**Table 1:** Each label in the data set along with its description.

*Data Label*	*Description*
Positive class	Annotators should give a positive label if the tweet describes feelings of pleasure, satisfaction, compliment, or recommendation. They are also advised to consider the tweet's subjectivity or objectivity and to annotate positive comparisons as Positive classes. Though positive emojis may be good indicators of positive sentiments, there are cases where the sentiment carried by the emoji is different from the context of the sentence that carries it resulting in uncertainties and disagreements.
Negative class	Annotators should give a negative label if the tweet describes feelings of disagreement, disapproval, complaint, worry, or hate. They are also advised to consider the tweet's subjectivity or objectivity. Also, they are to annotate unfavourable comparisons as negative classes.
Neutral class	The neutral label must be given for any text the annotator could not identify as positive or negative. Texts that are facts like reports, News, and general statements are to be annotated neutral. The tweet should contain a sentiment that does not justify a positive or negative class

**Table 2:** Language labels with its description.

*Language Labels*	*Description*
Monolingual Twi Dataset	Annotators should give a label monolingual if the tweet contains only Twi language words.
Multilingual Dataset	Annotators should give the label multilingual if the tweet contains Twi language words mixed with other languages.
Ghanaian Pidgin Dataset	Ghanaian Pidgin is characterized by different combinations of words, and annotators are to be able to label Pidgin by paying attention to words used in the language.

Quality Manual annotation (Fig. 2) is required for a comprehensive corpus and this is achieved if standard guidelines and training are provided and adhered to as in [9]. The guidelines used include the following:

## 4 Experimental Design, Materials and Methods

**Fig. 1:**
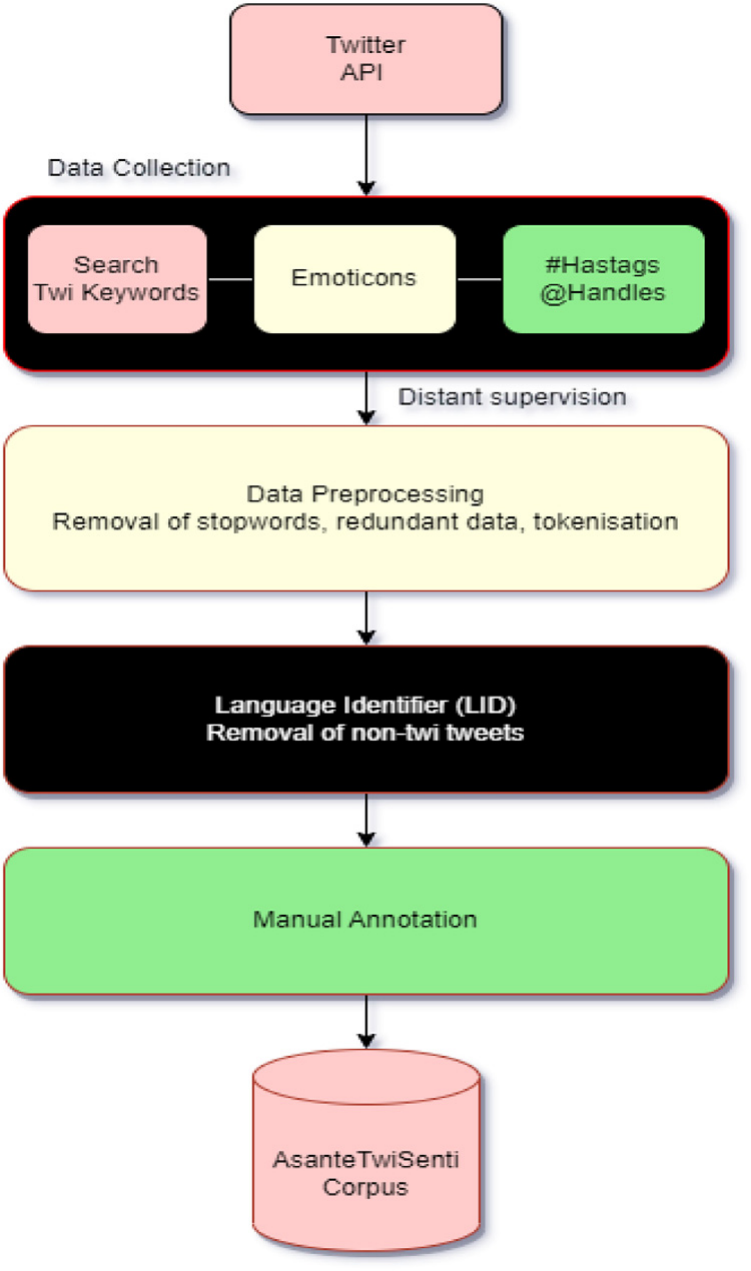
Data flow diagram for Twi Sentiment dataset construction.

In Fig. 1, an Asante Twi Tweets collection approach is shown, the Twitter API is queried with Twi stop words and emojis, hashtags, and handles of people in a query search in the Twi Language. The distribution of tweets into different classes is shown in Fig. 3. A word cloud representation of the most frequent words in the Twi tweets, Multilinqual tweets and Ghanaian Pidgin tweets datasets are shown in (Fig. 4, Fig. 5, Fig. 6). A Twi Sentiment Lexicon is shown in (Fig. 7).

**Fig. 2:**
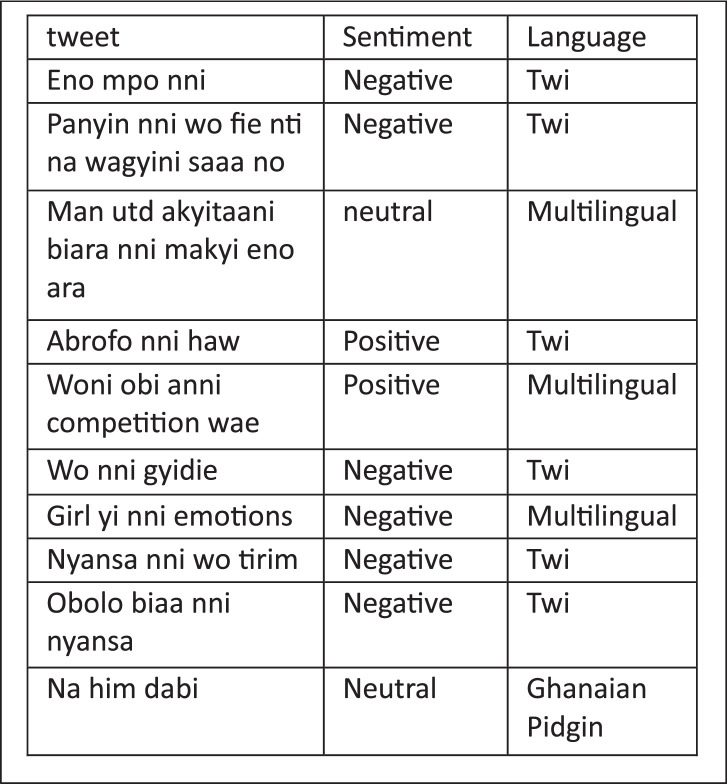
Sample annotated Tweets. This figure shows examples of annotated tweets from the Twi Sentiment dataset. Each tweet is labeled with a sentiment class (e.g., positive, negative, neutral) to demonstrate the dataset's structure and annotation approach.

**Fig. 3:**
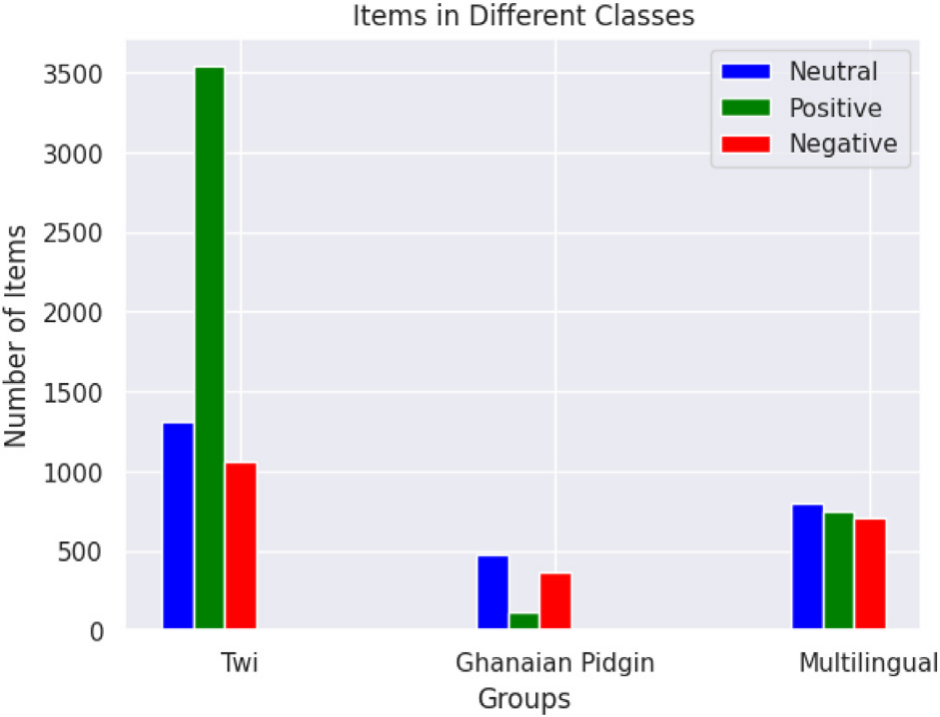
The distribution of tweets into different classes. This figure depicts the distribution of tweets across the three sentiment classes: positive, negative, and neutral. The chart highlights the class imbalance observed in the dataset and provides insights into the dataset composition.

**Fig. 4:**
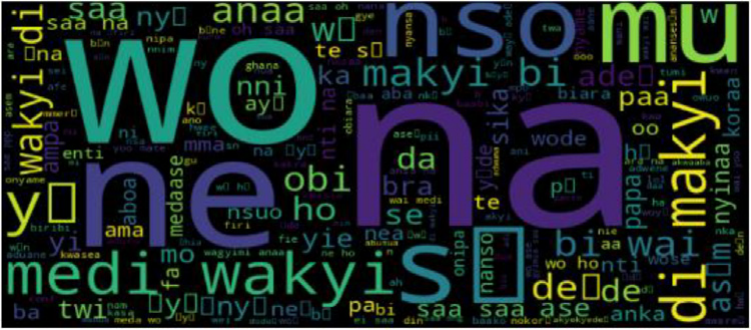
Twi Tweets word cloud. This figure presents a word cloud representation of the most frequent words in the Twi tweets dataset. The size of each word corresponds to its frequency, offering an overview of the linguistic features in the dataset.

**Fig. 5:**
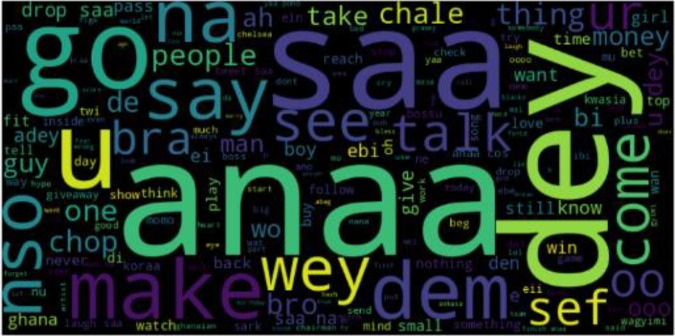
Multilingual tweets word cloud. This figure presents a word cloud representation of the most frequent words in Multilinqual tweets dataset.

**Fig. 6:**
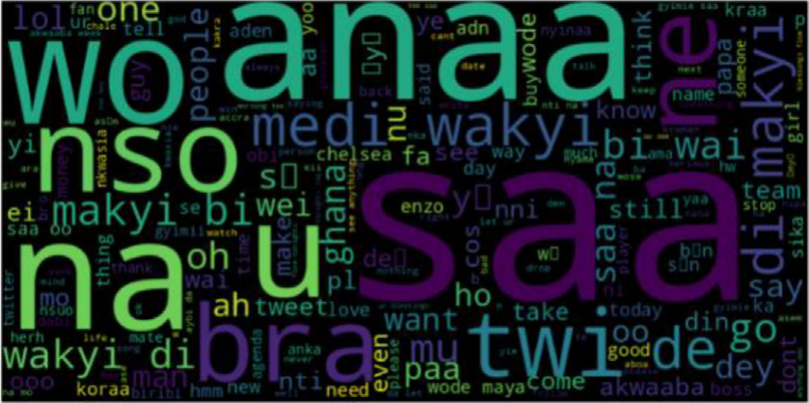
Ghanaian Pidgin word cloud. This figure presents a word cloud representation of the most frequent words in Ghanaian Pidgin tweets dataset.

**Fig. 7:**
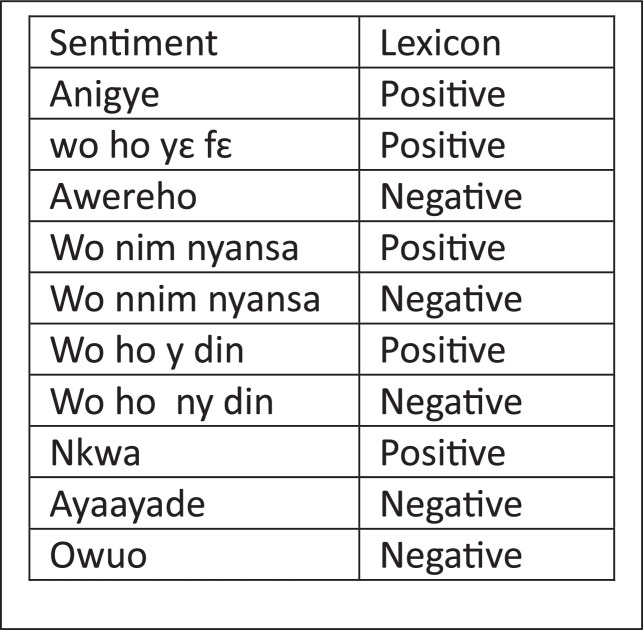
Twi Sentiment Lexicon visualization. It includes examples of sentiment-labeled words and highlights its distribution across positive, negative, and neutral sentiment categories.

This figure illustrates the steps followed in constructing the Twi Sentiment dataset. It highlights processes such as data collection, preprocessing, annotation, and validation, detailing how raw tweets are transformed into labeled datasets.

**Table:** 

*Data Label*	*Description*
Data Preprocessing	Redundant data removal is required for a comprehensive corpus construction. The dataset contains similar words, tweets written in other languages, these have to be filtered out. Hausa tweets and languages like Arabic, English, French, and Pidgin tweets are present in the dataset. Some of the search words in Asante Twi are also found in these languages. Irrelevant parts of the data such as duplicates, retweets, mentions, and trailing white spaces are also removed. Tweets that contain less than three words are maintained unlike in [3] because three words in Asante Twi can carry meaningful information. The challenge here is to remove all the redundant data to enhance the dataset quality. Twi language tweets need to be identified first to facilitate noise removal. A careful study of a tweet shows the presence of Tweet Identifier, search terms, @usernames and #hastags of users, date, time, and the tweet's location. To abide by Twitter terms and ensure privacy, user details are removed in the data preprocessing stage for the Privacy and Protection of Users.
Language Identification Detection(LID)	A Lexicon-based LID is used during language identification. Sentences of different languages are used to query a dictionary to check for sentence similarity. as used in [10], based on the words used in a tweet, tweets are identified as Twi or not. If the language detected is Asante Twi, a Python program writes to a file else it ignores the tweet, this helps to separate Twi sentences from the other languages as used in [11]. A cleaned and reduced dataset is achieved. Almost half of the dataset is given labels for machine learning.. The cleaner the dataset, the easier the annotation process. The resultant Dataset still had non-Twi words and these were removed manually as was done by [12].
Annotators	Three graduate students who are native speakers of the Asante Twi language from the linguistic department of the Kwame Nkrumah University of Science and Technology (KNUST) are recruited, and two native Asante Twi language speakers are also recruited as reviewers from the computer science department of the university to settle disputes in cases of ambiguity. Annotators are to abide by given guidelines and not to depend on their intuition only. For quality assurance, Reviewers are assigned to oversee and affirm the work of the annotators
Training of the Annotators:	Assigning Labels to opinions as positive, negative or neutral class by annotators works well if sentences are simple, straight to the point and concise , in cases where uncertainty exists as to which class the sentence belongs to, then based on [13], High-quality annotations can be achieved with clear and straightforward instruction,. If instructions are not given during the annotation, annotators are left to their intuition alone which may result in doubts and guesswork. They present an alternative way to achieve high-quality annotation by asking respondents to determine the target of a sentence and its sentiment in a semantic-role-based sentiment questionnaires.
Annotation Guidelines	The provided annotation guidelines focus on the classification of subjective tweets from Twitter. A subjective tweet may have a positive, negative, neutral emotion, opinion, or attitude. The guidelines given to the annotator define five classes: positive (POS), negative (NEG), neutral (NEU), multilingual, Twi, and Ghanaian Pidgin. The sentiment lexicons in AFFIN and NRC [14,15] are leveraged to build sentiment lexicons for the Asante Twi Language. Manual collection of Twi sentiment lexicons took place during the annotation process: by selecting common sentiment-bearing words which can be used to automatically classify tweets as positive and negative, as seen in [2].
Manual Annotation Tools	Annotation makes content more meaningful. Annotation Tools are useful, they simplify the process of labeling, marking, and tagging the parts in data. Selecting the right tools for annotation is very important as it improves efficiency. Some commonly used annotation tools include the following: Doccano, Annotate, Click Up, Drawboard, Lightag, and others. Lightag annotation tool designed by [8] helps manually label large datasets. It provides quality assurance through inter-annotator agreements with easy access to shared tasks on the platform. Before assigning Jobs to Annotators, a schema is created with the multiclass labels. The dataset is uploaded, given a schema, on which the annotators use to annotate the dataset as: Positive, Negative, Neutral, Ghanaian Pidgin, Multilingual, and Twi.
Inter-Annotator Agreement (IAA)	Reliable and trustworthy annotations are a good measure of consistent performance and a fair understanding of the annotators work. This can be tested by assigning the annotators similar labels. Similar results mean that the annotator's understanding of the annotation guidelines is similar and performance is accurate. Using Manual classification, machines learn human annotations to determine the overall polarity of texts. If two or more annotators cannot determine the class of a tweet, then a machine classifier cannot determine the class of such a text correctly. The Lightag tool automatically reported the IAA score of 0.52 on parts of the dataset. The IAA score shows the level of understanding of the annotators. Any score greater than 0.5 is medium and significant as proof of annotator's knowledge about the language and therefore their ability to deliver quality and accurate labels to each tweet. From above, 0.52 is a good score which implicate that the quality of the dataset is good and so useful as in [14]
Sentiment Lexicons	Sentiment lexicons are created by identifying the sentiment words in each tweet, A word list is created by annotators with their corresponding labels during the annotation. By leveraging the collected sentiment lexicons with the translated versions of the well-known English sentiment lexicons, AFFIN [15] we add up to the sentiment lexicon lists with more words.

## Limitations

Scarce digital data sources continue to be a challenge for many under-resourced languages due to the lack of appropriate keyboards, dictionaries, and digital tools specific to a Language. Special letters not present in English like ɛ, ɔ in Twi, are most of the time given symbols similar to the language orthography.

So ɔ is replaced by the symbol “)”, and ɛ is replaced by “3”. It is important to identify these to understand and annotate the tweets accordingly. Understanding the socio-cultural background of the Twi language is also fundamental to understanding user's tweets. Though emoticons can help in sentiment detection, their incorrect use can result in labeling controversies.

## Ethics Statement

The data presented in this paper is obtained using Twitter's services and by strictly adhering to their Terms of Service. All data is officially retrieved through Twitter APIs, in accordance with their Developer Agreement and Policy. As per Twitter's copyright policy, tweets may be owned by various entities. However, we have safeguarded the privacy rights of individuals in this dataset by removing each user's identity from the tweets and comments."

Automatically detected human sentiments vary depending on the domain and the people involved; so, we do not recommend this when making important decisions affecting individuals. Instead, it is useful for detecting broader sentiment trends on a large dataset.

## CRediT authorship contribution statement

**Mavis Sarah Gyimah:** Conceptualization, Methodology, Data curation, Formal analysis, Writing – review & editing. **James Benjamin Hayfron -Acquah:** Project administration, Supervision, Writing – review & editing. **Rose-Mary Mensah Gyening:** Formal analysis, Methodology, Writing – review & editing. **Michael Asante:** Project administration, Supervision, Writing – review & editing. **Umar Farouk Ibn Abdulrahman:** Writing – review & editing, Formal analysis. **Evans Kotei:** Writing – original draft.

## Data Availability

github repository.AsanteTwiSenti: A Sentiment Dataset of Ghanaian Asante Twi Tweets in a Multilingual Context. (Original data)
